# Career sacrifice unpacked: From prosocial motivation to regret

**DOI:** 10.3389/fpsyg.2022.874142

**Published:** 2022-09-16

**Authors:** Jelena Zikic

**Affiliations:** School of Human Resource Management, York University, Toronto, ON, Canada

**Keywords:** career sacrifice, work relationships, family relationships, career decision making, career success and career outcomes, career shocks, career self-management

## Abstract

In the ever more uncertain career context, many individuals engage in a form of career sacrifice (CS) at some point in their career journey; that is, giving up of certain career goals/actions or reshaping career decisions to accommodate specific work or life demands. This conceptual paper unpacks CS as an important yet little explored dimension of career decision making. Specifically, the paper examines possible triggers of CS as well as the diverse nature of CS, ranging from short-term (usually minor) type of sacrifice to more significant and long-term sacrifice. We explore the context of this type of career decision making, specifically the intersection of work and non-work-related triggers and conclude by discussing possible work and non-work outcomes both at the individual as well as organizational level. CS outcomes range from enhanced career self-management and relational benefits to positive organizational contributions, but at times can also lead to regret. Areas for future research are identified, especially exploration of demographic and more macro level variables as possible moderators in CS decisions. Future theoretical development of CS is discussed too.

## Introduction

We commonly discuss contemporary careers as becoming more boundaryless, offering opportunities beyond organizational, national, and even work-life boundaries (e.g., Arthur, 2014). Career studies have seen plethora of writing highlighting these increasing possibilities, leading to more global and varied career journeys. This move has also been accompanied by a very self-directed approach to career management where agency seemed to dominate over the role of career structures (Wilhelm and Hirschi, 2019). This was an appealing move, as it seemingly allowed the individual to take control, to plan and navigate one’s career based on personal preferences and an ever-evolving life structure. This perspective has also been criticized and is often countered by a call to bring back the boundaries into careers discourse (Gunz et al., 2007). This opposing view highlights perhaps the more realistic side of careers, namely that they inevitably evolve amid many different boundaries and even more likely barriers (Baruch and Vardi, 2016). Thus, one way to consider both career perspectives simultaneously may be to focus on the kinds of decisions and (re)negotiations that career actors may have to undertake to manage their careers.

**FIGURE 1:**
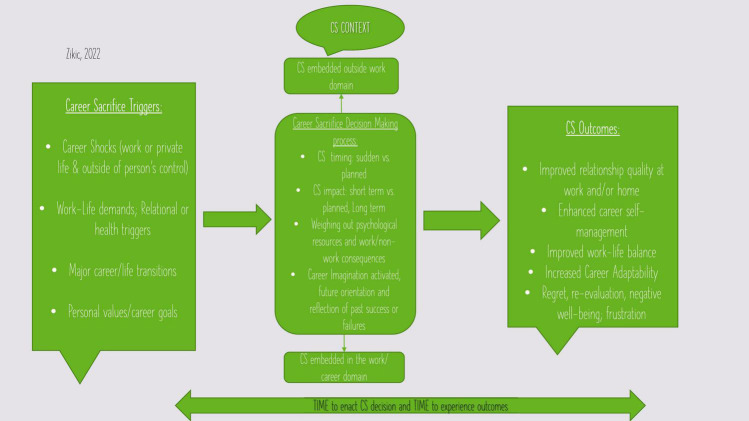
Career sacrifice unpacked.

Given the current climate of increasing uncertainty (i.e., living under the pandemic regimes) and rapidly evolving labor market characteristics and demands (Snyder, 2016; Spurk and Straub, 2020), we constantly struggle to further restructure and re-negotiate our careers and work-life boundaries. As a result of this continuously changing career context, many career actors are forced to give up certain aspects of their careers or to seriously reshape their career plans, future career action, or even how they understand career success (Baird et al., 2021). This giving up of certain career goals/actions or reshaping of our decisions to accommodate specific work or life challenges, can be understood as career sacrifice (CS) (see Table 1 and Figure 1). Thus, while CS can be triggered from either work or non-work domain, it can also be motivated by a combination of those factors. CS is commonly part of our decision making, yet it is little understood career action that we rely on to manage our careers in the context of ongoing life and career challenges.

**TABLE 1:** Career sacrifice: Triggers, types and context.

CS triggers	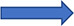	Internal (personal/internal decision): issues related to one’s work values/career plans or decisions driven by personal/family needs; health or care giving issues. External (externally driven): labor market changes/downsizing; major career transition such a move/migration or career change due to external factors. Mix of internal and external factors.
CS types	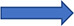	Short term/temporary (e.g., work-life solution): forgoing a vacation; working longer hours; staying on a particular project longer; forgoing a promotion; forgoing study/development plans; postponing a new role. Long term/permanent (impact on passion, purpose, identity): giving up one’s preferred line of work/occupation; retiring early; taking multiple roles/type of work (i.e., survival jobs) instead of one’s preferred occupation; having to learn new skills/engage in a new occupation.
CS context	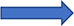	Organization/work role related Work-family based Relationship focused (work and non-work) Personal values/goals Intersection of the above domains.

## The nature of career sacrifice: Long-term vs. short-term career sacrifice

As mentioned above, CS involves a decision to give up fully or partially one’s immediate career course as to accommodate another life or career related action. CS highlights the intricate relationship between work and non-work domains, as well as an important ability to self-regulate one’s career. By engaging in CS, we postpone a goal or forgo an opportunity, which is an important exercise of self-control (Shane and Heckhausen, 2019; Milyavskaya et al., 2021) and a form of career self-management.

CS decisions usually combine and consider the more objective career characteristics (e.g., promotions, objective career success dimensions) with subjective ones (e.g., personal satisfaction, wellbeing) (Gunz and Heslin, 2005). Thus, CS is also intricately linked to emotions in our careers and as such presents a much less understood aspect of our work lives (Kidd, 2008). CS can also happen in the context of commonly experienced career challenges such as job loss, career change or involuntary career transitions, all leading to intense emotional experience and a need to engage in some type of CS. Thus, CS is an integral part of our careers and related decision-making, yet it has not been explored in the current career literature. CS may be triggered by either external forces (i.e., structural barriers, innovation/change in the labor market/occupational field, migration transition) or specific internal ones (i.e., personal/family decision or injury/health related issue); but also due to a mix of internal (i.e., desire to move) and external issues (i.e., labor market conditions).

CS is distinguished from classic career decision making, as addressed in vocational psychology, as it does not necessarily involve a list of promising alternatives, or even as much reliance on “true reasoning” (Parsons, 1909; Gati and Kulcsár, 2021). Rather, CS combines context as well triggers often in the form of internal and external career shocks (Akkermans et al., 2018), and can occur at any life/career stage. Importantly, we are focusing specifically on the “giving up” rather than methodical planning as in classic career decision making literature. Thus, some major career decision has already been put in place (i.e., choosing one’s occupation/organization, career path), and CS is the process that changes that course of action to some extent and in relation to the context (i.e., internal or external triggers). Lastly, while classic career decision making is seen as logical, rational and a conscious cognitive process, CS decision making is likely the opposite, namely not always orderly or logical, and sometimes may even be guided by intuition for example (Gati and Levin, 2012).

The timing of CS may also vary and is not specifically linked to any particular career or life stage. Thus, CS can happen early on in one’s career or it can be triggered by forces in later career stages and even into retirement. Early career stage, typically correlating with younger age, may mean less experience within a particular line of work for example, and less investment of resources in the particular career path; thus, it may possibly be easier for a career actor to sacrifice some aspect of one’s work life at an early career/life stage (Lent and Brown, 2020). Yet, with more work experience, major career investments (typically in later life stage), also larger network of contacts, sacrifice (i.e., giving up of some of those investments) decisions may be harder to enact (e.g., Shane and Heckhausen, 2019). Specifically, it may be much more difficult to give up or forgo any aspect of one’s well-established career and to change professional identity as well (Zikic and Richardson, 2016). Thus, career stage may possibly moderate some of the CS decisions. Similarly, one may also wonder whether any demographic factors may impact CS decisions (see future directions for possible moderators of this type).

Finally, a decision to sacrifice some aspects of our work may also involve some type of “inaction” (Verbruggen and De Vos, 2020); this may be considered a passive form of sacrifice (Impett et al., 2005). For example, putting our studies/further development on hold, or waiting to apply for a specific position when the time is ripe, or even taking time off work (i.e., pausing one’s career) to attend to other issues outside of work. Yet, a more active form of CS may involve taking deliberate action that allows one to alter the current course. For example, having to search for a new role/job or having to learn a new skill or even to fully change one’s occupation due to some external triggers (i.e., technical innovation; major career transition) that prevented one from continuing a desired career path. In the next section we address in more detail the types of CS and its triggers.

### Long-term career sacrifice

CS can be intricately related to renouncing on our passion or a sense of purpose (Ho et al., 2021), to which we have special attachment; in which case, a sacrifice can mean a long-term change that affects one in some major ways. This type of CS may have a major impact on our career wellbeing and can involve negative emotions or even regret. Long-term CS may involve having to renounce and forgo practicing in a beloved profession (i.e., no longer able to be a doctor, lawyer, professor etc.) or a trade, or no longer be able to use a particular skill (i.e., due to change or innovation). Professional identity of individuals in these groups is usually very strong, and the most salient aspect of their sense of self (Zikic and Richardson, 2016), thus having to sacrifice this aspect of one’s identity is a major change in the self. Long-term CS may also lead to pursuing an alternative career and fully abandoning one’s original profession (Gati, 1993).

Another example of long-term CS may also be due to career adversity encountered in the local labor market, due to migration transition or an international move (e.g., resulting in lack of credential recognition, discrimination, or a lack of opportunities locally) (Zikic and Richardson, 2016). In these cases, CS may mean that the career actor sacrifices previous education and work experience in favor of a new beginning in a more developed economy or even safer life for the whole family. In these situations, CS may also mean settling for a simple survival job or even renouncing on the world of work completely and dedicating oneself to the family/non-work domain fully. This extreme type of CS clearly presents one end of the continuum in terms of degree and permanency of sacrifice.

In other cases, individuals may engage in CS that involves renouncing on an important skill or part of their career profile, which can still have long term negative consequences for the career actor. For example, a particular skill/ability to work on a specific machine or use a particular tool or software may no longer be needed, due to more advanced techniques or updated systems (i.e., new technology/innovation). These events may have made one’s skill redundant or obsolete and will lead to a long-term sacrifice; perhaps leading to a completely new career course or a major career change (Baruch and Vardi, 2016; Akkermans et al., 2020).

The above examples of a long term, that is more permanent type of CS will often set-in motion major career decision making process (i.e., career self-management), typical of a major career transition (Lent and Brown, 2020). Long term CS may also take longer time to enact, as it is a major change that requires preparation and often exploration of various career options. It may mean that a career actor must completely re-construct his or her identity (Zikic and Richardson, 2016) following a major CS. It is much harder, leaving one’s beloved line of work, than it is putting a temporary hold one’s career plans due to caregiving/non-work obligations for example, which is a more short-term and often minor type of CS, as discussed next.

### Short-term career sacrifice

As noted above, CS can also be considered minor, often shorter term, and more temporary in nature. For example, one may be forced to simply postpone or put on hold current career plans; the sacrifice may be limited to one aspect of our work lives and be short lived. A common situation would be postponing going up for a promotion due to heavy family obligations (i.e., raising a young family) or temporarily taking time off from work to accommodate various non-work or caregiving needs. In these cases, the CS decision may also lead to positive spillover effect (Greenhaus and Powell, 2006) and enrich either family or work domain. In these examples, the career actor is aware of the benefits of waiting, putting on hold certain plans in a short term and the sacrifice may also be experienced as more positive in nature. Specifically, when CS is made on one’s own volition due to family or outside of work obligations, the individual may feel more in control of their own career decision, and there is hope that in the near future, one can still continue on the desired career path. Short term sacrifice is typically seen in many career stages and individuals may engage in these decisions on regular basis. These short-term sacrifice decisions may also be triggered by the external context or events, such as the current pandemic context whereby many career actors had to postpone certain plans, such as hold their next career move or postpone further development/training plans.

At other times, especially in early career, focusing for example on further training and development may be beneficial in terms of one’s future career growth, thus forcing one to forgo other work opportunities at the time. CS may also involve relinquishing an international work opportunity or simply travel opportunities in the interest of a bigger career plan or due to completing one’s planned educational journey. Other times, organizations may also require certain level of experience (i.e., longer time in a role), thus preventing one from certain promotional opportunities. Relatedly, another type of short-term CS may have to do with forgoing holidays/time off, giving up certain perks (i.e., bigger office/organizational vehicle); or postponing a move to a new role due to commitment to a particular job/project or a team. As the nature of the short-term CS is often temporary, the career actor can clearly see or imagine a different future and opportunities (i.e., desired career plans) becoming reality, post CS; thus, leading to a more hopeful and perhaps positive experience overall.

Finally, short vs. long term types of CS may also involve different underlying motivations. Having to look for a new career course or a learning opportunity as one’s skills may no longer be valuable, is typically based on an avoidance motivation (e.g., avoiding the unemployment situation) (Impett et al., 2014). This type of sacrifice may involve avoiding negative career experiences, stress, or frustration, and in the long run may even lead to lower career wellbeing or even regret. On the other hand, the scenario where CS was less long-term and seen as a temporary solution, is often based on a positive motive; thus, more approach related motivation and emotions such as the ability to spend more time with the family or focus on one’s studies/career opportunity as opposed to avoidance of potential negative circumstance (Kidd, 2008).

## Career sacrifice in context

CS decisions can be situated in the context of one’s work and/or outside of work domains (e.g., family/partner, caregiving, hobby, international opportunity) (see Table 1). Thus, CS must be understood at the intersection of various life domains; focusing on the interdependence that exists between those domains and social relationships that characterize them (Impett et al., 2005). Relational dynamics in the context of CS is especially dominant as careers do not develop in a vacuum but are rather influenced by social context and relationships that surround them (e.g., Higgins, 2001). In fact, CS is perhaps even more commonly triggered as many individuals are part of dual-career couples and many also have multigenerational caring responsibilities. While work-family literature is strongly influenced by the conflict perspective (e.g., Lapierre and McMullan, 2016), there has also been growing interest in the extent to which experiences in one role improve the quality of life in the other role, thus in the enrichment or positive spillover perspective (e.g., Fisher et al., 2009). Essentially, engaging in the CS decision, can be seen as an antecedent to reducing conflict for example or supporting the enrichment view. Specifically, the CS decisions often consider psychological investment in either role and a possibility where one could adjust perhaps by forgoing certain career or family/life plans or even completely disengaging from certain roles—all geared toward improved career, life satisfaction and wellbeing. Thus, the CS decision may be related to the contextual characteristics of the work domain and how these may be “adjusted” or changed in order to accommodate some outside of work role for example. One can go as far as saying that CS is almost always a negotiation related to the work-life decision and typically as antecedent to the enrichment paradigm (Lapierre et al., 2018).

CS may also develop in the context of our personal values, that is to be internally centered. It involves choices between equally valuable and important options; thus, to make a CS decision, one requires consideration and strong adherence to one’s own values (i.e., work, family values, ethical principles, even spiritual, and cultural values). Cultural values and expectations may also shape our CS decisions; for example, the importance assigned to the work domain may vary depending on the society (Baruch and Vardi, 2016). Cultural values may also influence how we make career decisions in the context of organizations. For example, in some societies where individuals experience high power distance, we may assume that the career actors may be much more hesitant to make certain types of sacrifices that involve, for example, accommodating non-work-related issues. Thus, CS may lead to pursuing perhaps the less desirable option for the individual, while it may be for the greater good of the team/organizational and perhaps supported by societal values. Similarly, being part of individualistic vs. collectivistic societies may also promote certain types of CS (i.e., relational/collectivist goals vs. pursuing own career goals). As a result, one’s image and reputation in the context of work may also be affected, depending on the type of CS that one pursues.

## Outcomes of career sacrifice

CS can simultaneously evoke both positive and negative feelings and outcomes for the self and others (see Table 1). While CS can be related to positive outcomes such as freeing up time for other important roles (i.e., non-work roles), or even pursuing other equally important choices (i.e., education/career opportunity), it may also have a very negative impact on the individual career actor. For example, depending on the identity salience (i.e., very career-oriented individuals), some types of CS may lead to frustration, re-evaluation of one’s decision and even strong sense of regret that may last a while (Budjanovcanin et al., 2019). CS can also evoke extremely positive self-evaluations by self and others, as it shows one’s ability to put one’s preferences on hold or even forgo certain opportunities for the benefit of others (i.e., relational focus) or other important work or non-work goals (Impett et al., 2005). Thus, the outcome dynamic is complex and can be seen on a continuum and varying from very pro-social and positive to more negative feelings; these far-reaching consequences can impact one’s career future, individuals’ wellbeing as well as quality of relationships.

### Continuum of career sacrifice outcomes: From pro-social outcomes to regret

The most obvious benefit of CS is related to the prosocial aspect of this behavior, that is, benefiting someone or some other greater good in the career or life of the actor. The ability to forgo an immediate career related self-interest to promote the wellbeing of others demonstrates not only willingness to sacrifice but most importantly, self-control and behavioral type of sacrifice (Righetti et al., 2020).

Secondly, when the CS decision is embedded in the context of non-work domain, it can be seen as “pro-relationship” behavior; it is positively associated with relationships outside of work as well as personal wellbeing. In this case, CS has a positive spillover effect (Greenhaus and Powell, 2006). CS may also signal long-term relationship commitment and lead to greater relationship satisfaction in the future. In this case, the CS is often driven by communal motivation, that is a desire to provide care and increase the wellbeing of others (Righetti et al., 2020). It can increase positive feelings toward the self—that is feeling proud for doing the right thing, as well as providing care and responding to the needs of others. This type of prosocial behavior may also increase self-efficacy and self-esteem offering evidence to the self and others that we are capable and receptive to the needs of others.

Yet, on the other side of the CS outcome continuum, the same CS situation may also lead to negative feelings or the experience of regret. Regret is a possible outcome of CS and presents an intense emotional experience based on realizing that one should have taken a different decision (Righetti and Visserman, 2018), likely not have sacrificed in this case. When CS leads to this negative outcome, it may range from a constant feeling of conflict or guilt for having sacrificed an aspect of one’s career and at the same time seeking satisfaction from enacting or satisfying the other role, the initial trigger for CS. This type of negative outcome may be long lasting and affect one’s general work or life satisfaction and wellbeing (Budjanovcanin and Woodrow, 2022).

### Outcomes of career sacrifice in the organizational context

In the context of organizational life, CS may also signal one’s willingness to postpone his or her own career goals for the greater communal/organizational good (see Table 2). This can in turn lead to improved work relationships and likely, a more positive self-image and reputation for the one enacting CS. Moreover, showing willingness and even more the actual CS behavior in the context of one’s team, such as leadership or team member role related sacrifice, will provide a powerful signal/role modeling for the followers and colleagues in general (Van Knippenberg and Van Knippenberg, 2005). CS behavior in the context of the leadership role may also set a norm for others and show dedication to a bigger cause or to a particular organization. CS in the context of one’s work group also promotes a more collectivistic attitude. That is the opposite of the more individualistic career discourse that is often part of the contemporary careers (Baruch and Vardi, 2016).

**TABLE 2:** CS in organizations: Managerial implications.

CS type/context		Implications
CS in the work-life domain: leading to part-time, temporary leaves, or more permanent leave	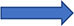	Gender considerations; managerial support and understanding for the CS decision; pre-post CS conversations and role arrangements. Clarify expectations; Post CS/return accommodations (i.e., returnship).
CS embedded in the existing role: role change, learning and development leave, postponing promotion. Forgoing other options due to commitment to the current project/team. Giving up perks/time off for the benefit of the team/org.	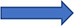	Understanding and appreciation for the CS type and the need for it. Organizational reward/gratitude task vs. relationship conversations both pre-post CS.

### Individual level career outcomes

A CS decision likely represents a “discontinuity,” a type of career interruption; there may be a need to re-construct one’s career to some extent and seek continuity pre- and post-CS situation. Thus, when engaging in any kind of CS decision-making, career actor may benefit from focusing on the future orientation and career planning. Yet, in some cases, due to an immediate and strong external trigger, CS may also have to be made on an ad hoc basis without much consideration for future career plans (Lent and Brown, 2020; Gati and Kulcsár, 2021). For example, one may be forced into CS by migration, unexpected family decision/accident or due to major political or economic triggers. Both internal and external triggers may lead to sudden CS decision and perhaps less desired outcomes for the career actor as well as post CS regret. Regret may be temporary or longer-lasting, but it always involves imagining and wishing that the CS decision turned out differently; or that one had more choices or more time to make the CS decision (Budjanovcanin et al., 2019). The consequences of CS can thus be seen in career steps non-taken. Thus, regret may be more likely due to sudden CS decision or a context that triggered an immediate CS without much consideration or prep.

Overall, however, the decision-making that accompanies CS can enhance one’s ability to reflect upon the past, focus on the present, or plan for the future (Baird et al., 2021). In each of the cases discussed above, CS requires a career self-management outlook, in this case usually done independently of any career coaching or counseling advice. Career self-management precisely involves the type of activities that one must engage in when contemplating CS, while also considering its contextual and other consequences (Gati and Kulcsár, 2021). For example, it involves an ongoing assessment of values, goals, identity clarification, or understanding one’s life and career priorities (Wilhelm and Hirschi, 2019). CS can also be a trigger for a major career change or a transition and must involve important and complex career decision-making. Finally, the individual career actor engaging in CS is also learning to tolerate and manage career uncertainty and exercise flexibility in decision-making, which requires optimism and self-reliance (Eva et al., 2020).

## Implications for practice

CS is common in contemporary careers and managers will have to manage them accordingly (see Table 2). On the managerial side, providing understanding, support, and possible accommodation for the variety of CS decisions, both pre-and post-CS, may be critical. Managers must also provide a balance of task-orientated vs. relationship-oriented conversations, that show gratitude to employees who have decided to engage in CS for the benefit of the organization and build a positive image and role modeling for the future. Relatedly, organizations may also have to manage the return to work for some of these employees. On the organizational side, having these career related conversations both pre-and post-CS will assist with workforce planning as well as setting realistic expectations on either side. Overall, patience and holistic understanding, and the ability of the organization to offer a transitional period of some kind may be important ways that organizations can assist those post CS or who are engaging in CS.

## Implications for research

In further exploring and empirically testing the CS phenomena, researchers should especially focus on the degree of CS as well as triggers coming from different life domains. That is, what kind of implications do minor vs. major CS decisions have on the decision maker and his/her future career success? Equally important would be a longitudinal focus to understand how career decisions of this type influence one’s career path pre and post sacrifice. Depending on the triggers from various contextual domains, researchers should also study some of the proposed outcomes of CS, for example improved relationship quality or enhanced work-family balance. To what extent do CS decisions lead to enrichment or spillover in one or the other domain? It would be important to also conduct qualitative research into the actual experiences of sacrifice and especially during major career decision making, how individuals cognitively engage in weighing out pros and cons of CS and understanding when do career actors may instead experience regret. Finally, long term vs. short term regret may also have different effects on the individual career actor. Finally, future research should also include emphasis on the demographic factors and explore whether gender, age, socioeconomic status, career stage and other such variables in fact moderate the likelihood that someone will engage in the CS decision. It would also be important to know whether some structural issues or macro level variables (i.e., political/social/economic triggers) can also moderate the type of CS decisions; similarly, investigating the role of certain cultural values to prevent vs. promote CS in the work vs. family context may be another avenue for future research.

## Career sacrifice in the context of the pandemic uncertainty

Due to the current pandemic measures and resulting economic uncertainty and crisis, many individuals have lost jobs or were forced to close businesses that previously brought them not just financial benefits but also great personal satisfaction (Rugaber, 2020). These individuals have to cope with both loss and sacrifice, and for some this may mean considering a new occupation, perhaps only survival job, or looking at re-training and starting from scratch. Thus, in the current context, CS may have more profound meanings and similarly to strong external triggers like migration and fleeing one’s home country, pandemic related CS may be long-lasting and involve negative emotions, such as regret, loss or frustration.

CS in the context of the pandemic may also be linked to the work-family domain; such sacrifice due to pandemic context may lead to leaving a job in order to take care of one’s children or elderly parents. CS in the context of the pandemic carries additional meanings; specifically, the uncertainty about the post pandemic career landscape, inability to plan for future career, as well as often very negative feelings such as helplessness, frustration, and regret for having to close business for example, or lose a job that one much enjoyed doing.

## Future research on career sacrifice

In addition to career stage considerations mentioned above, one may also expect gender to also play a role as an important demographic factor in CS decisions. Research suggests that perhaps due to multiple roles that women still take both at work and at home, they may in general be more likely to sacrifice especially when the CS decision is triggered from a non-work domain (Sullivan and Mainiero, 2008; Cerrato and Cifre, 2018). It is still the case that in many societies, women typically take on more care giving responsibilities and those non-work roles may have stronger identification and meaning for women compared to men (Cerrato and Cifre, 2018). Yet, as many societies take on more egalitarian roles in the family and at home, this may also be changing to some extent, even if at a slower rate. Thus, future research should examine the role of gender in various CS decisions. Finally, socioeconomic status as well as cultural norms may also play a role in CS decisions. Yet, given the lack of research on these more macro type influences as part of the CS context, we can only speculate that with lower socioeconomic status for example, individuals may experience greater economic pressures (i.e., unstable jobs perhaps as well as having to take more roles at home); thus, the dynamism of CS decisions for this group may be more pronounced and financial need may take precedence over other values or career related needs (Thompson and Subich, 2006). More macro type variables, such as cultural values as mentioned above, may also affect how we make CS decisions (Leung et al., 2011); for example, more collectivistic societies may give precedence to family and relational types of triggers while high power distance context may perhaps put unique pressures on workers and one’s need to engage in specific work-related sacrifice. These may be some fruitful avenues for future research, as these more macro level and sociological factors may add complexity to understanding societal as well as economic basis for how individual level sacrifice decisions are made.

## Future theoretical development of career sacrifice domain

CS falls within a relational perspective, which abandons the commonly seen dualism in career studies between structure vs. agency (i.e., as in Giddens’ structuration theory; Mayrhofer et al., 2007); instead, here we assume that individuals and context cannot be disconnected but are always embedded and conceived in relation to one another. From this stance, CS meaning making is situated in the nexus between agency and structure (Cohen and Duberley, 2021).

Cohen and Duberley’s (2021) recent concept of career imagination may form a potential source of inspiration for the future theoretical development of CS from this more balanced perspective. Cohen and Duberley (2021) take on a relational perspective and build on structuration theory to develop the concept of career imagination, i.e., how individuals think about their working lives. Their concept contains three dimensions: first dimension is related to perceptions of enablement and constraint, the second dimension is time, and highlights how career imagination is always contextually and temporally situated; while the third-dimension identity, but not purely individual but also socially situated.

Yet, while CS concept can benefit and further develop in the context of career imagination, it also differs from it in the following: CS concerns an actual decision whereas career imagination is about imaging (im)possibilities. Yet, both career imagination and CS have to do with career meaning making situated in context and time. The meaning individuals attach to the world and their position in it guide their perceptions of what is (im)possible and also their related career actions (Cohen and Duberley, 2021). Career imagination and its three dimensions may thus form the basis for all CS decisions. Through career imagination career actors imagine and contemplate CSs that are appropriate and possible. For instance, going into early retirement in order to open up jobs for younger employees may in early exit cultures feel as a right and possible thing to do even if one still would love to remain active. Career imagination may also change how career actors perceive a career decision over time. What seems to be an optimal decision and the natural thing to do at a particular moment in time, may over time transform into a CS because the actor’s imagination of possible and appropriate behavior has changed. For instance, women who gave up their career to take care of the children might not have seen that as a sacrifice at the time. But with changing values on motherhood and increased labor market participation of women, they might start to imagine that other career paths might have been possible and come to perceive that decision as a sacrifice.

Thus, a more balanced and relational perspective on CS leads to different avenues for future research and allows for needed complexity in this type of decision making. In a relational perspective on agency and structure, context is no longer introduced as a pregiven variables “out there” (e.g., labor market conditions) separate from the individual (Janssens and Steyaert, 2019). Also, in a relational perspective, time is considered to be socially embedded. Future research from this balanced and relational perspective might tackle questions on what career actors see as (im)possible and (in)appropriate, how that is embedded in space and time, and how this imagination shapes their perceptions and action career action related to CS. With our future theoretical development of CS, we aim to broaden our scope on CS from triggers and consequences of a particular CS decision at a particular moment in time toward CS as a career meaning making process.

## Conclusion

Career decision making is a complex process and continues in some capacity over the course of one’s career (Gati and Kulcsár, 2021). Yet, many of our career decisions involve a type of CS, that is, giving up of certain career goals/actions or reshaping of our decisions to accommodate for our work or life challenges. While general career decision making has been studied before, sacrifice as one important dimension of career decision making has not been sufficiently explored nor considered in current careers research. This conceptual paper explores possible triggers, types, context, and outcomes of CS across a life span. The goal was to start to unpack what CS involves conceptually and identify avenues for future empirical research. Future research should also explore possible moderators, especially demographic factors that may impact the extent or willingness to sacrifice. This paper highlighted the difference between short term, that is commonly made CS vs. long term or what is termed major CS. Importantly, CS decisions are situated at the intersection between work and life domains. Thus, contextual basis for sacrifice will commonly involve issues and challenges that revolve around either a work focused situation or life outside of work or a combination of both. Finally, various types of outcomes of CS were outlined, with the emphasis on the relational as well as career self-management related benefits.

## Author contributions

The author confirms being the sole contributor of this work and has approved it for publication.
